# *Schistosoma mansoni* infection is associated with changes in gut microbiota in preschool age children in Albertine Region, Uganda

**DOI:** 10.21203/rs.3.rs-9768290/v1

**Published:** 2026-05-25

**Authors:** Andrew Edielu, Chiao-Wei Lo, Patrice A. Mawa, Emily L. Webb, Alison M. Elliott, John Kelvin Mugerwa, Gloria Oduru, Jacent Nassuuna, Gloria Kakoba Ayebazibwe, Monika Struebig, Jennifer F. Friedman, Amaya L. Bustinduy, Martin J. Holland

**Affiliations:** Immunomodulation and Vaccines Theme, MRC/UVRI & LSHTM Uganda Research Unit, Entebbe, Uganda; Department of Clinical Research, London School of Hygiene and Tropical Medicine, London, United Kingdom; Immunomodulation and Vaccines Theme, MRC/UVRI & LSHTM Uganda Research Unit, Entebbe, Uganda; MRC International Statistics and Epidemiology Group, London School of Hygiene and Tropical Medicine, London, United Kingdom; Department of Clinical Research, London School of Hygiene and Tropical Medicine, London, United Kingdom; Immunomodulation and Vaccines Theme, MRC/UVRI & LSHTM Uganda Research Unit, Entebbe, Uganda; Immunomodulation and Vaccines Theme, MRC/UVRI & LSHTM Uganda Research Unit, Entebbe, Uganda; Immunomodulation and Vaccines Theme, MRC/UVRI & LSHTM Uganda Research Unit, Entebbe, Uganda; Immunomodulation and Vaccines Theme, MRC/UVRI & LSHTM Uganda Research Unit, Entebbe, Uganda; Department of Clinical Research, London School of Hygiene and Tropical Medicine, London, United Kingdom; Center for International Health Research, Rhode Island Hospital, Providence, United States; Department of Clinical Research, London School of Hygiene and Tropical Medicine, London, United Kingdom; Department of Clinical Research, London School of Hygiene and Tropical Medicine, London, United Kingdom

**Keywords:** Schistosomiasis, gut microbiota, preschool age children

## Abstract

Current understanding of gut microbiota alterations during helminthiasis is largely derived from experimental models, often focusing on a narrow range of metrics. This study investigates the structural and functional shifts in the gut microbiome associated with *Schistosoma mansoni* infection in a paediatric cohort. We conducted a cross-sectional study of preschool-aged children (12–47 months) comparing *S. mansoni*-infected individuals (56) to uninfected controls (57). Microbial DNA was extracted from stool samples and sequenced via the Illumina MiSeq v3 platform targeting the V4–16S rRNA region. Diversity was assessed through alpha (Chao1, Simpson, Shannon) and beta (UniFrac and Bray-Curtis distance) metrics. Functional potential was predicted using PICRUSt2 mapped against the KEGG database. The infected group (median age 36 months) exhibited significantly higher alpha diversity and species richness compared to uninfected peers (median age 26 months). Beta diversity analysis confirmed distinct microbial clustering between the two groups (p-value = 0.001). Notably, *S. mansoni* infection was characterized by the proliferation of pro-inflammatory taxa and a concomitant depletion of short-chain fatty acid (SCFA) producers. Functional modeling indicated a significant downregulation of metabolic pathways involved in energy metabolism and SCFA biosynthesis. *S. mansoni* infection is associated with profound structural and functional dysbiosis in preschool-aged children. The depletion of SCFA producers and altered metabolic pathways suggest that infection may impair host nutritional status and influence the parasite’s lifecycle, necessitating further longitudinal investigation.

## Introduction

Schistosomiasis is caused by a trematode of the genus *Schistosoma (S.)*, and affects approximately 240 million worldwide with pre-school aged children (PSAC) bearing a high burden (Exum. et al., 2019). The lifecycle of schistosomes involves a freshwater snail as an intermediate host and a vertebrate definitive host. While adult *S. mansoni* and *S. japonicum* reside within the host’s portal and mesenteric veins, the continuation of the parasite's life cycle relies on the successful translocation of eggs across the vascular and intestinal barriers. This migration into the intestinal lumen is a critical step for environmental shedding (Nation. et al., 2020). Being highly immunogenic, the eggs elicit an inflammatory response, accompanied by development of a granuloma which aids its transit to the intestinal lumen.

Granuloma formation is an immune-mediated process modulated by both parasite and host factors, such as the gut microbiota (Costain. et al., 2018). With approximately 10 times more bacterial cells and 100 times the genomic content than their human host, gut microbiota influences host metabolism, immune function, and other processes (Thursby. & Juge., 2017). Various factors such as host genetics, nutritional status, diet and infections affect gut microbial homeostasis (Anwar. et al., 2021). Perturbations in the composition of gut microbiota in comparison to the community structure in healthy individuals is known as dysbiosis (Petersen. & Round., 2014). Changes in gut microbial communities are typically evaluated using alpha and beta diversity metrics: alpha diversity quantifies richness and evenness within individual samples, while beta diversity assesses the compositional dissimilarity between distinct groups or environments (Cassol et al., 2025).

All three major species of *Schistosoma* – *S. mansoni, S. japonicum* and *S. haematobium* – have been associated with alterations in gut microbiota in human and mouse experimental studies. The trend of existing evidence points to increased beta diversity coupled with decreased alpha diversity (Ajibola. et al., 2019; Cortés. et al., 2019; Jenkins. et al., 2018; Zhao. et al., 2019). While shifts in microbial community structure are evident, the functional consequences on host morbidity and parasite fecundity warrant further exploration.

Studies from mouse experimental models indicate that the gut microbiota affects the pathophysiology of schistosomiasis through alteration of granuloma formation and subsequent fibrosis in the liver and the intestinal tissues (Stark. et al., 2023; Zhang. et al., 2020). Depletion of gut microbiota through antibiotic treatment in mice is associated with reduced egg excretion due to defective granuloma formation that is attributed to alterations in immune responses (Holzscheiter et al., 2014). Because the immune response to eggs trapped in host tissues is the basis of pathology in schistosomiasis, the extent of successful egg passage is likely to have clinical implications for the host. In a mouse experimental model, pre-infection microbiota composition was associated with *Schistosoma* worm burden and egg count, a scenario attributed to increased susceptibility of mice with certain microbial communities to colonisation by schistosomes (Corté. et al., 2020), again highlighting the role of the gut microbiota in perpetuating the schistosome life cycle. However, some studies have also reported no changes before and after treatment of *S. haematobium* (Kay. et al., 2015). Furthermore, a critical analysis of existing literature is complicated by significant heterogeneity in reported metrics of microbial community structure and function, coupled with a paucity of consistent human data. Extrapolating these findings to paediatric cohorts is particularly challenging, as the childhood gut microbiome is a dynamic ecosystem, undergoing successional changes influenced by age-specific nutritional and dietary shifts. In this study, we investigate association of *S. mansoni* infection with gut microbial composition and accompanying functional pathways that may be impacted and make inferences on clinical consequences for the host.

## Materials and methods

### Study design, setting and population

This study used a cross-sectional design to compare gut microbiota structure and function of *S. mansoni*-infected PSAC with an uninfected comparison group. Both groups of participants were recruited from Buliisa and Hoima districts in the Albertine Region of Uganda from April 2021 to February 2023. The study area is a rural setting with a significant population of the two districts dwelling in fishing villages along the shores of Lake Albert which provides economic livelihood and water for household use. For the infected group, stored baseline samples and data from the *Praziquantel for children under age four years: A Phase II PK/PD driven dose finding trial (PIP trial)* were used (Webb et al., 2021). Uninfected PSAC aged 12–47 months whose parents/caretakers provided consent were recruited from an area of lower schistosomiasis endemicity within the same districts as PIP trial participants.

### Study procedures, sample collection and processing

Fifty-six baseline stool samples from the PIP trial were selected from a collection of 354 available samples were randomly selected while fifty-seven uninfected PSAC were separately recruited recruited using an analogous approach to the PIP trial as per published protocol (Webb et al., 2021). At a designated location in the community, participant’s age was verified from the parent/caretaker and using child health card where available, followed by obtaining written informed consent and clinical assessment.

*S. mansoni* infection status was confirmed by serial testing using urine Point of Care Circulating Cathodic Antigen (Schisto POC-CCA^®^, Rapid Medical Diagnostics, South Africa), stool Kato Katz (KK) technique (Bärenbold et al., 2017) and urine Circulating Anodic Antigen (CAA), which was quantified using an up-converting phosphor-lateral flow circulating anodic antigen (UCP-LF CAA) assay (Leiden University Medical Centre, Netherlands) at a cut off at > 2ρg/ml for infection (Sousa. et al., 2019). Infected participants were positive by all three tests while the comparison group were negative by the same tests.

Participants whose eligibility was confirmed provided a final stool sample from which two aliquots were taken in 2ml cryovials and immediately stored in liquid nitrogen at the field research station in Buliisa district. These were transported in the liquid nitrogen to MRC/UVRI & LSHTM Uganda Research unit for storage at −80°C.

### DNA extraction and 16S rRNA library generation

Microbial DNA from faecal samples and extraction kit controls were extracted using QIAamp^®^ Fast DNA Stool Mini Kit (Qiagen Diagnostics GmbH, Dusseldorf, Germany). Extracted samples were then transported to the London School of Hygiene and Tropical Medicine where DNA quantity and yield was determined by Qubit^®^ 2.0 Fluorometer (Thermo Scientific) before adjusting the final concentration to 10ng/ul in a total volume of 20 ul (except for extraction controls in which the concentration was below detection limits). DNA amplification through Polymerase Chain Reaction (PCR) was then done as follows. 15 μL of Phusion Master Mix (New England Biolabs) was added to 2 μM of forward and reverse primers, with 10ng template DNA or 2 ul extraction control eluate. Thermal cycling consisted of initial denaturation at 98°C for 1 minute, followed by 30 cycles of denaturation at 98°C for 10 seconds, annealing at 50°C for 30 seconds, and elongation at 72°C for 30 seconds, and final extension at 72°C for 5 minutes. Amplicon libraries were generated using TruSeq^®^ DNA PCR-Free Sample Preparation Kit (Illumina, USA) following manufacturer's recommendations and index codes were added. The library quality was assessed on the Qubit^®^ 2.0 Fluorometer (Thermo Scientific) and Agilent Bioanalyzer 2100 system. The library was sequenced on an Illumina NovaSeq platform to generate 250 base pair (bp) paired end reads. Paired-end reads were assigned to samples based on their unique barcode and truncated by trimming the barcode and primer sequence, then merged using FLASH (V1.2.11, http://ccb.jhu.edu/software/FLASH/) to yield raw tags (Magoc. & Salzberg., 2011). Quality filtering on the raw tags was performed using fastp (version 0.23.1) to obtain high-quality clean tags (Bokulich. et al., 2013). To obtain the effective tags, chimera were removed by comparing the raw tags with the 16S sequences in the Silva reference database using UCHIME Algorithm (Edgar. et al., 2011).

### Bioinformatics and statistical analysis

Denoising of the clean sequences was done in DADA2 software, and the resulting Amplicon Sequence Variants (ASVs) were annotated against the Silva 138.1 database to yield taxonomic classifications and abundance data. Microbial community richness and evenness were compared using Shannon, Simpson and Chao1 indices as measures of alpha diversity. The ASVs were also used for constructing an evolutionary diagram and an Unweighted Pair-group Method with Arithmetic Mean (UPGMA) cluster tree to determine the phylogenetic relationship between the taxa in each sample. Based on the evolutionary diagram, unweighted UniFrac distances were calculated using QIIME2 software, and weighted UniFrac distances obtained by adjusting the measurements for relative abundance. UniFrac distances from the two groups were compared using Principal Coordinates Analysis (PCoA) and Non-parametric Multidimensional Scaling (NMDS). Bray-Curtis distances between the two groups were also compared to measure similarity based on ASV abundance. MetagenomeSeq analysis and independent t-tests were used to determine differences in microbial community structure between the groups, while Linear Discriminant Analysis Effect Size (LEfSe) was employed to identify significant functional differences. PICRUSt2 software was used to predict microbial functional potential based on the abundance of 16S rRNA gene sequences (Douglas. et al., 2020). This was achieved by inferring genomic content through phylogenetic placement and linking the results to the Kyoto Encyclopedia of Genes and Genomes (KEGG) database (Kanehisa. et al., 2023).

## Results

### Baseline characteristics

A total of 113 stool samples were collected. A total of 56 were from PSAC infected with *S. mansoni* (Sm Pos) while 57 were from uninfected PSAC (Sm Neg). Median age was 36 months (IQR 24–42), and 26 months (IQR 20–34) for the infected and uninfected groups respectively. There were 29 males (52%) and 36 males (63%) in the infected and uninfected groups respectively. No significant difference in sex distribution between the groups was observed (p-value = 0.221). There was also no difference in haemoglobin concentration and weight-for-height z-scores across the groups (p-value = 0.273 and p-value = 0.162 respectively). However, *S. mansoni* infected PSAC had lower weight-for-age z-scores (p-value = 0.003) and higher height-for-age z-scores (p-value < 0.001) compared to the uninfected PSAC. The overall weight-for-Height z-score (WHZ) was not significantly different between groups (p-value = 0.162).

### Descriptive Statistics

**Figure 1:**
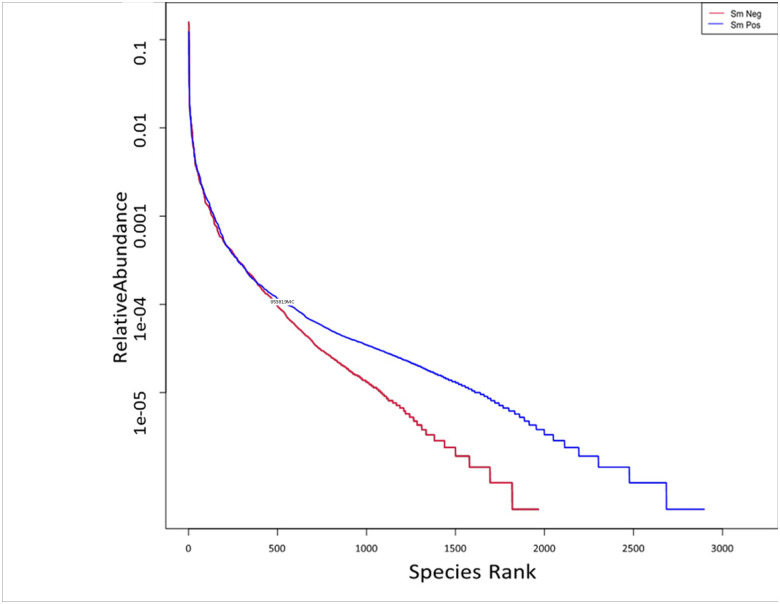
Rank abundance curve for gut microbial communities from PSAC with *S. mansoni* infection (Sm Pos) and uninfected PSAC (Sm Neg). The steeper curve (Sm Neg) depicts less richness and evenness.

One hundred thirteen samples were analysed. After quality control filtering a total of 7,830,324 reads were generated with an average read depth per sample of 62,719 reads (range; 37,868–107,742). A total of 26 phyla, 47 classes, 111 orders, 166 families and 286 classified and 26 unclassified genera were identified from the V4-amplicon sequence reads. The infected group also showed higher abundance of species (Fig. 1). The distribution of shared and unique ASVs between the two groups is illustrated in the Venn diagram **(Supplementary Figure S1)**.

### S. mansoni infected PSAC show increased alpha diversity of gut microbiota

**Figure 2:**
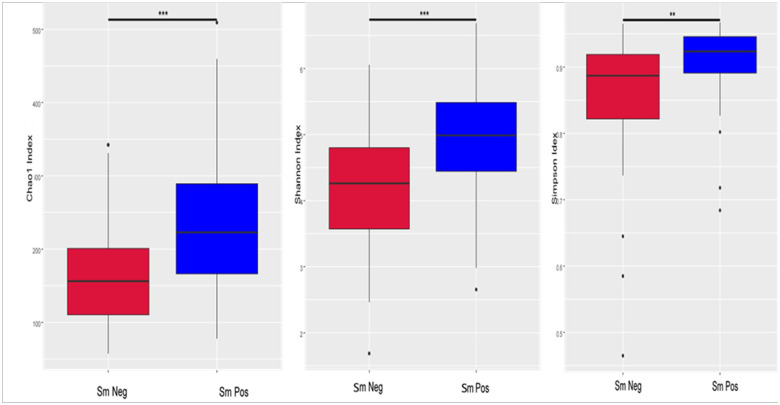
Box plots comparing alpha diversity indices of PSAC with *S. mansoni* infection (Sm Pos) and uninfected PSAC (Sm Neg).

The comparison of alpha diversity indices is presented below, with higher diversity in the infected group across the three metrics. (Fig. 2).

### Increased beta diversity in S. mansoni infected group

**Figure 3:**
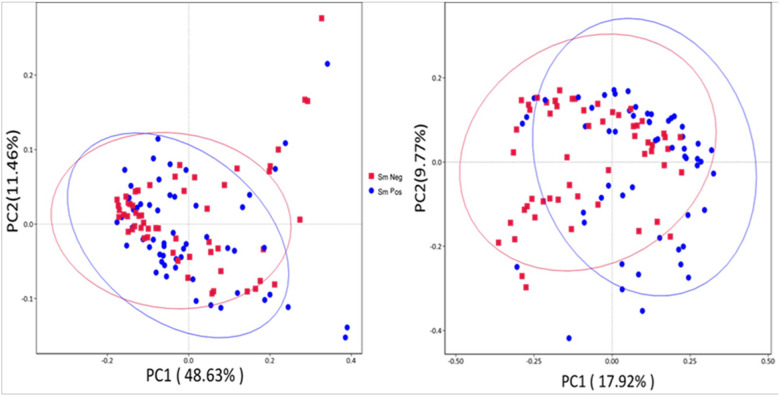
PCoA plot of Weighted (left) and unweighted (right) UniFrac distance matrix representing beta diversity patterns of taxa in infected children (Blue/Sm Pos) and uninfected children (Red/Sm Neg). Each point represents a sample.

Weighted UniFrac distance was clustered using the Unweighted Pair-group Method with Arithmetic Mean (UPGMA) to generate a cluster tree in **Supplementary Figure S2**. PCoA ordination was used to explore differences in microbial community structures between the two groups based on UniFrac distances. In the PCoA plot of unweighted UniFrac, *S. mansoni* infection (PC1) explains 48.83% of the total observed variance while sample differences between the two groups (PC2) explains 11.46% of the total variance in the observed abundance between infected PSAC and uninfected PSAC (Fig. 3). In addition to PC1 and PC2 explaining a majority (combined 60.29%) of the variance, noticeable differences in microbial communities between the two groups is observed based on the PCoA plots.

**Figure 4:**
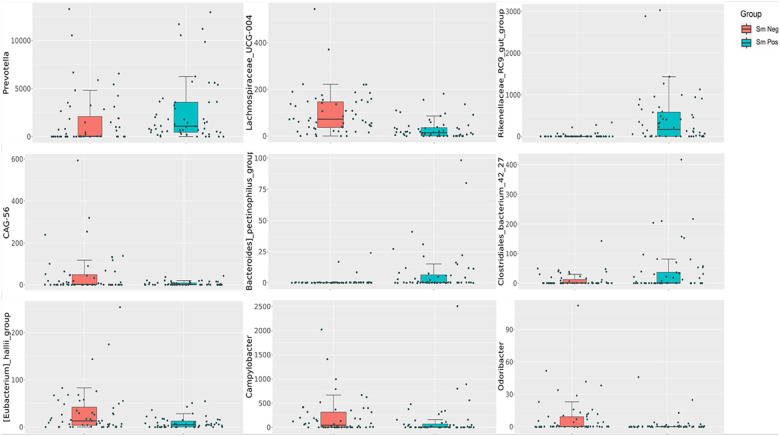
Boxplots showing genera with differences in abundance between *S. mansoni*-infected PSAC (Sm Pos) and uninfected PSAC (Sm Neg).

To determine if the observed differences are statistically significant, non-parametric tests, Analysis of similarity (ANOSIM) and Permutational multivariate analysis of variance (PERMANOVA) were used. There was a statistically significant difference between the microbial communities of infected versus uninfected participants according to ANOSIM (R = 0.08, p-value = 0.001). R-values in ANOSIM range between − 1 to + 1, with a positive value indicating that the intergroup differences is greater than the within-group difference. This finding is in line with PERMANOVA, which is based on the Bray-Curtis distance matrix (F = 3.76, p-value = 0.001). A t-test was done to determine genera and species with significant differences between the infected and uninfected PSAC. The taxa with significant differences are shown in Fig. 4 and **Supplementary Figure S3**. In the infected group, there was proliferation of *Prevotella*, *Bacteroides* (pectinophilus group), *Rickenellaceae* RC9, and *Clostridiales_bacterium*_42_27, accompanied by depletion of *Odoribacter*, *Lachnospiraceae* UCG004, CAG-56, *[Eubacterium]_halii*_group and *Campylobacter*.

### Biomarker differences between groups

Linear discriminant analysis Effect Size (LEfSe) was used to assess differences in microbial biomarker profiles between the two groups (Segata. et al., 2011) as shown in **Supplementary Figure S4**. *S. mansoni* infection was associated with proliferation of *Oscillospirales, Prevotella* and *Oscillospiraceae*, and depletion of *Bacilli, Enterobacteriaceae, Lactobacillales, Lachnospirales* and *Lachnospiraceae*.

### Functional differences in microbial communities

A total of 4421 orthologues were identified, of which 3920 were shared while 338 and 163 were unique to the infected and uninfected groups respectively **(Supplementary Figure S5)**. The affected orthologues and their significance are shown in **Supplementary Figure S6** and Table 2 respectively.

## Discussion

Interest in understanding the link between helminths and gut microbiota has been gaining momentum, with the goal of making inferences on clinical and diagnostic parameters. In this study, we investigated the association of *S. mansoni* infection with structural and functional features of gut microbiota in pre-school aged children (PSAC).

Our findings revealed that *S. mansoni* was associated with increased alpha diversity, as measured by Chao1, Simpson and Shannon indices, when compared to uninfected participants. Similarly, beta diversity was increased in the infected group when considering overall species abundance, UniFrac and Bray-Curtis metrics. There was also increased abundance of *Rickenellaceae, Bacteroides pectinophilus group, Clostridales* and *Prevotella*, with reduction in populations of *Odoribacter, Lachnospiraceae UCG-004, Eubacterium hallii group, CAG-56* and *Campylobacter*. LEfSe analysis demonstrated statistically significant increases in abundance of *Prevotella* and *Oscillospirales*, with reduction in *Bacilli, Enterobacteriaceae, Lactobacillales* and *Lachnospirales*.

Our findings align with the general observation of changes in gut microbiota during helminth infections. In an experimental mouse model, it has been demonstrated that *S. mansoni* infection is associated with changes in gut microbiota at day 28 and day 50 post-infection. They reported increased beta diversity but reduced alpha diversity at day 50 (Jenkins. et al., 2018). Another study showed increased beta diversity in *S. japonicum*-infected mice compared to the uninfected controls. Alpha diversity was increased in BALB/c mice but reduced in C57BL/6 mice (Zhao. et al., 2019).

Considering the longevity of schistosomes (Warren. et al., 1974), mouse experimental models may not accurately represent longer-term impact of infection considering that children in areas of high schistosomiasis endemicity such as the Albertine Region of Western Uganda have a potential to be infected prior to the first birthday (Stothard. et al., 2013). Compared to uninfected children, one study in children 2–15 years reported with *S. mansoni* infection marginal increases in Chao1, observed species abundance and phylogenetic diversity, with accompanying decrease in Shannon index, all of which were not statistically significant (Schneeberger. et al., 2018). In Zimbabwean PSAC with *S. hematobium*, infection status and intensity had a significant effect on composition of bacterial and fungal genera. No direct metrics of alpha diversity were reported (Osakunor. et al., 2020). Another study in children with *S. haematobium* infection reported higher abundance and diversity of gut microbiota, and significant proliferation of five species of *Prevotella* (Kay. et al., 2015) in line with our findings. Together with other studies with similar methodology, present a picture of altered composition in gut microbiota by schistosomes, despite their diverse findings.

There is evidence that schistosomes actively influence gut microbiota changes. (Floudas. et al., 2019). Manipulation of gut microbiota by schistosomes could be geared towards perpetuation of the worm’s lifecycle. Mice in which gut microbiota has been depleted through antibiotic treatment have reduced stool egg count. This is supported by evidence that successful egg transit is facilitated by granuloma, whose formation is immune-dependent and influenced by gut microbiota (Holzscheiter et al., 2014; Schwartz. & Fallon., 2018).

We report that *S. mansoni* infection is associated with proliferation of Oscillospirales, *Prevotella* and *Oscillospiraceae*, and depletion of *Bacilli, Enterobacteriaceae, Lactobacillales*, Lachnospirales and *Lachnospiraceae*. Due to their proximity to the gut mucosa, commensal *Clostridia* play a key role in modulating immune function and other metabolic processes (Lopetuso. et al., 2013). Similarly, *Prevotella* is not only critical in training of the gut immune responses through production of colonic Th17 (Huang. et al., 2020), but also modulation of gut inflammatory responses (Iljazovic. et al., 2021). Moreover, proliferation of *Prevotella* has been associated with colitis, accompanied by reduction in SCFAs (Iljazovic. et al., 2020) whose roles include gut immune homeostasis and reinforcement of epithelial integrity (Venegas. et al., 2019). *Lachnospiraceae* also plays a role in maintenance of epithelial barrier, as do *Bacilli* that have been shown to upregulate expression of Tight Junction (TJ) genes. Their depletion may therefore reduce integrity of gut epithelium, with similar effect as proliferation of *Prevotella*. The effect of *S. mansoni* on the gut microbiota therefore appears geared towards an enhanced inflammatory phenotype and compromised gut epithelial integrity, a combination of which aides egg passage to the lumen (Costain. et al., 2018).

Despite the above evidence of the effect of schistosomes on gut microbiota composition, the functional pathways through which the observed changes affect host function remain poorly understood. Our findings demonstrate downregulation of pathways involved in starch breakdown, biosynthesis of amino acids and other secondary metabolites such as SCFAs. The Embden-Meyerhof pathway responsible for conversion of glucose to pyruvate was also downregulated. Reduction in pyruvate is in line with findings of reduction in acetic acid by *Strongyloides stercoralis* (Nguyen. et al., 2022) and may be a key pathway in helminthic infections.

SCFAs are produced by commensal bacteria in the colon and are important in neuro-signalling, attenuation of lipopolysaccharide-induced inflammation and increased absorption of glucose in the muscles. They are also precursors for lipid synthesis in the liver (He. et al., 2020). It is therefore possible that the cognitive deficits (Ezeamama. et al., 2017) and the malnutrition observed in schistosomiasis (McGarvey., 2000) could originate from disruption of the SCFA pathway from gut microbiota.

It should be noted that microbiota is influenced by multiple environmental and host factors such as diet, prevailing nutritional status and host genetics. Moreover, 16S amplicon sequencing relies on a small section of the genome (hypervariable region 4) compared to whole genome sequencing methods. Infected and uninfected participants also had slight variation in anthropometric parameters, a factor that can influence gut microbial homeostasis. Despite these challenges, our study provides unique insights across a wide spectrum of structural and functional gut microbiota metrics during *S. mansoni* infection and uses an uninfected comparison group.

Future work should aim for closer matching of participants by age, gender and geographical location by possibly recruiting infected and uninfected participants from the same household to control for such variables as diet. Comparison of individuals before and after treatment of *S. mansoni* infection can also provide better insight of temporary association with infection. Methods such as shotgun metagenomic sequencing should also be employed for more comprehensive assessment of gut microbial community structure and function. Concurrent metabolomics analysis would be insightful in linking any observed changes in microbial communities to actual profile of metabolites such as SCFAs.

In conclusion, *S. mansoni* infection is associated with structural and functional changes in gut microbiota. Proliferation of pro-inflammatory taxa potentially promotes egg transit hence perpetuating schistosome life cycle and could also mitigate pathology by influencing granuloma formation. Depletion of short chain fatty acid producers may have adverse consequences for host metabolism, immune function and nutrition.

## Supplementary Material

This is a list of supplementary files associated with this preprint. Click to download.

- SupplementaryFigureS1.png
- SupplementaryFigureS2.png
- SupplementaryFigureS3.png
- SupplementaryFigureS4.png
- SupplementaryFigureS5.png
- SupplementaryFigureS6.png
- SupplementaryTableS1.png

## Figures and Tables

**Table 1:** Baseline characteristics of *S. mansoni* infected and uninfected PSAC in Albertine Region of Western Uganda

Sex (Male)	*S. mansoni* uninfected PSAC (n = 57)	*S. mansoni* infected PSAC (n = 56)	p-value
	36 (63%)	29 (52%)	0.221
Age in months (Median (IQR))	26 (20, 34)	36 (24, 42)	< 0.001
Haemoglobin concentration (g/dl) (Median (IQR))	11.2 (10.1–12.1)	10.8 (10.0–11.8)	0.273
Haemoglobin levels, n (%)	12 (21.1)	11 (19.6)	0.261
Moderate Anaemia (7.0–9.9 g/dL)	12 (21.1)	20 (35.7)	
Mild anaemia (10.0–10.9 g/dL)	33 (57.8)	25 (44.7)	
Normal haemoglobin (≥ 11.0 g/dL)			
Weight-for-Height z-score (WHZ) (Median (IQR)	−0.6 (−1.4, 0.0)	0.4 (−0.2, 1.4)	0.162
Weight-for-Age z-score (WAZ) (Median (IQR)	0.7 (0.1–1.4)	0.1 (−0.6, 0.6)	0.003
Height-for-Age z-score (HAZ) (Median (IQR)	−2.1 (−2.7, −1.0)	−0.5 (−1.5, 0.1)	< 0.001

**Table 2:** KEGG orthologues with significant functional differences between S. mansoni infected PSAC and uninfected PSAC.

KEGG Orthologue	Name of orthologue	Functional pathway and significance	Association with S. mansoni
K08303	U32 family peptidase	Pathway map05120, Epithelial cell signalling in Helicobacter pylori infection, unknown role in humans	Upregulated
K07024	Sucrose-6-phosphatase	ap00500 pathway for starch and sucrose metabolism (catalyses breakdown of sucrose to glucose). map01110 pathway for biosynthesis of secondary metabolites.	Downregulated
K15634	2,3-bisphosphoglycerate-dependent phosphoglycerate mutase	map01230 pathway for biosynthesis of amino acids Embden-Meyerhof pathway (convert glucose to pyruvate)	Downregulated
K01624	Fructose-bisphosphate aldolase, class II	map00010 pathway for glycolysis / gluconeogenesis Embden-Meyerhof pathway (convert glucose to pyruvate)	Downregulated
K00656	Formate C-acetyltransferase	Pyruvate, propanoate and butanoate metabolism	Downregulated

## Data Availability

Anonymized metagenomic sequence data is available in the National Centre for Biotechnology Information Sequence Read Archive (NCBI SRA), accession number PRJNA1463197.

## Key findings

1. *S. mansoni* infection is associated with profound structural and functional dysbiosis in preschoolaged children.
2. Increased alpha diversity in the *S. mansoni* infected group
3. Increased abundance of species in the *S. mansoni* infected group
4. *S. mansoni* is associated with depletion of short chain fatty acid producers
5. *S. mansoni* is associated with downregulation of pathways involved in short chain fatty acid production and energy metabolism
